# In silico structural homology modeling and characterization of multiple N-terminal domains of selected bacterial Tcps

**DOI:** 10.7717/peerj.10143

**Published:** 2020-11-03

**Authors:** Mohammed Alaidarous

**Affiliations:** Department of Medical Laboratory Sciences, College of Applied Medical Sciences, Majmaah University, Majmaah, Saudi Arabia; Health and Basic Sciences Research Center, Majmaah University, Majmaah, Saudi Arabia

**Keywords:** Toll/interleukin-1 receptor domain, Bacteria, N-terminal domain, Immunity, Homology modeling

## Abstract

Several bacterial pathogens produce Toll/interleukin-1 receptor (TIR) domain-containing protein homologs that are important for subverting the Toll-like receptor (TLR) signaling cascades in hosts. Consequently, promoting the persistence and survival of the bacterial pathogens. However, the exact molecular mechanisms elucidating the functional characteristics of these bacterial proteins are not clear. Physicochemical and homology modeling characterization studies have been conducted to predict the conditions suitable for the stability and purification of these proteins and to predict their structural properties. The outcomes of these studies have provided important preliminary data for the drug discovery pipeline projects. Here, using in silico physicochemical and homology modeling tools, we have reported the primary, secondary and tertiary structural characteristics of multiple N-terminal domains of selected bacterial TIR domain-containing proteins (Tcps). The results show variations between the primary amino acid sequences, secondary structural components and three-dimensional models of the proteins, suggesting the role of different molecular mechanisms in the functioning of these proteins in subverting the host immune system. This study could form the basis of future experimental studies advancing our understanding of the molecular basis of the inhibition of the host immune response by the bacterial Tcps.

## Introduction

In mammalian hosts, the innate immune system works as the first line of defense against microbial invasion. An immune response is induced upon recognition of the bacterial conserved pathogen-associated molecular patterns (PAMPs) by the hosts’ pattern recognition receptors (PRRs). TLRs are predominant PRRs that recognize various bacterial PAMPs. Upon PAMPs recognition, TLRs oligomerize, initiating an intracellular immune signaling cascade. TLR oligomerization brings both the ligand binding and the cytoplasmic domains into close proximity, which is followed by the recruitment of various cytoplasmic adaptor proteins. The cytoplasmic protein interactions are known to be mediated by the conserved cytoplasmic TIR domains present in the TLR receptors and cytoplasmic adaptor proteins. These interactions activate downstream transcription factors including the nuclear factor-κB (NF-κB) that upregulate the expression of multiple inflammatory mediators (Kawai & Akira, 2010; Ve, Williams & Kobe, 2015).

Microbial pathogens have been shown to counter host innate immune defense pathways through molecular mimicry and evasion of the immune response (Elde & Malik, 2009). Several bacterial proteins with immune evasive properties have been detected in a range of Gram-negative and Gram-positive bacteria. These proteins are TIR domain-containing proteins (Tcps) that are structurally similar to several mammalian host Tcps, and are crucial for bacterial subversion of the TLR signaling cascades. It was reported that the TIR domains are involved in protein-protein interactions with TLR receptors and/or the cytoplasmic adaptor proteins (Rana et al., 2013; Ve et al., 2012). In a previous study, more than 200 TIR homologues were identified in a wide range of bacterial species, including *Brucella* species, *Escherichia coli* and *Salmonella enterica* serovar Enteritidis (Newman et al., 2006). A subsequent work identified a Tcp in the non-pathogenic *Paracoccus denitrificans* (Low et al., 2007). However, the role of Tcps in non-pathogenic bacteria remains poorly understood. More recently, bacterial Tcps have been detected in *Yersinia pestis*, *Staphylococcus aureus*, *Helicobacter pylori*, *Yersinia pseudotuberculosis*, *Enterococcus faecalis* and *Pseudomonas aeruginosa* (Askarian et al., 2014; Imbert et al., 2017; Kaplan-Türköz, 2017; Kraemer et al., 2014; Nörenberg et al., 2013; Patterson et al., 2014; Rana et al., 2011). Bacterial Tcps are approximately 230-310 amino acids long containing TIR domains with primary sequences varies between 150-200 amino acids. In bacterial Tcps, the TIR domain can be located in either the N-terminal or the C-terminal region while the remaining regions are highly variable (Patterson & Werling, 2013). Understanding the exact molecular mechanism of Tcp-dependent bacterial evasion strategies for subverting the host immune system is important as the number of reported bacterial Tcps is rising.

The molecular functions and structural characteristics of various TIR domains from mammals, bacteria and plants have been the focus of several studies (Ve, Williams & Kobe, 2015). The available data suggest that microbial Tcps may function as dimers. However, the molecular mechanism of the Tcps dimerization is not clear (Alaidarous et al., 2014; Kaplan-Türköz et al., 2013; Ve et al., 2012; Ve, Williams & Kobe, 2015). Several studies suggested that domains other than the TIR domain (including the Tcps N-terminal domains), either from microbial or host Tcps, are involved in the Tcps dimerization, protein-protein interactions and/or binding to phosphoinositides in the cell plasma membrane (Alaidarous et al., 2014; Askarian et al., 2014; Kaplan-Türköz et al., 2013; Ve, Williams & Kobe, 2015; Xiong et al., 2019). Therefore, it is important not to focus on microbial TIR domains as sole players in microbial host immune subversion. Investigating the molecular mechanism of the full-length proteins and their N-terminal domains (NTDs) will provide a clearer understanding of the mechanisms involved. However, studies have shown that the solubility and stability of full-length Tcps decreases in solution (Alaidarous et al., 2014; Patterson et al., 2014; Salcedo et al., 2013). In this study, we use in silico approaches to determine the physicochemical and structural characteristics of multiple NTDs of selected bacterial Tcps.

## Materials and Methods

### Prediction and analysis of the primary amino acid sequences

**Figure 1:**
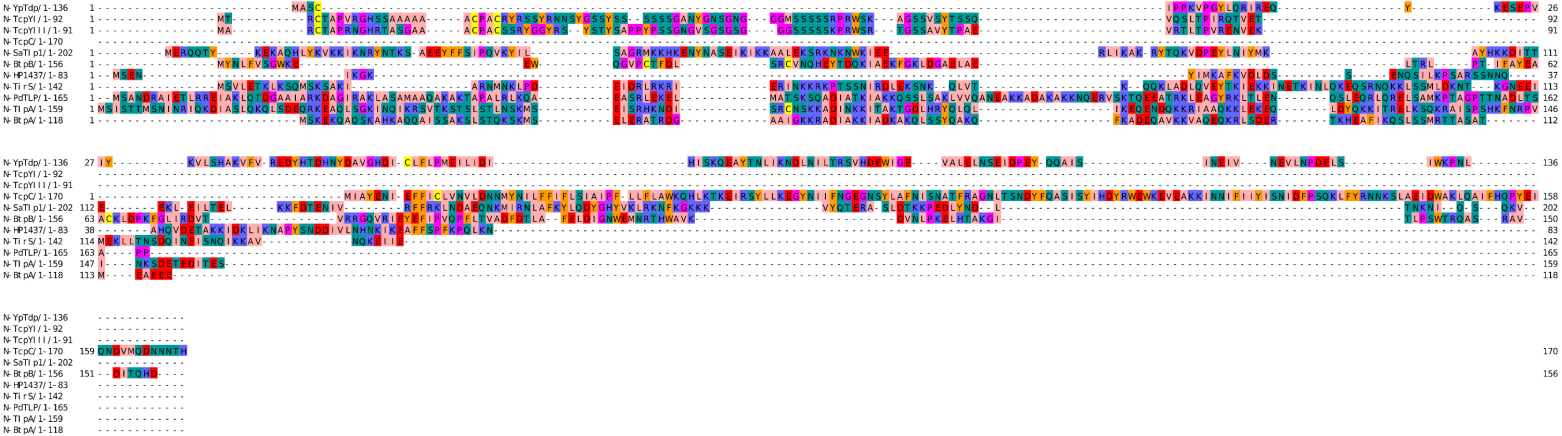
Sequence alignment of the NTDs of selected bacterial Tcps. The sequence alignment estimated by Clustal Omega and viewed using Jalview.

The bacterial Tcps sequences used in this study were retrieved from the Universal Protein Resource (UniProt) (http:/www.uniprot.org) in the FASTA format for analysis. These proteins include the Tcps from the Uropathogenic *Escherichia coli* (TcpC, UniProt ID A0A0H2V8B5) (Yadav et al., 2010), *Brucella melitensis* (BtpA, UniProt ID Q8YF53) (Alaidarous et al., 2014; Kaplan-Türköz et al., 2013), *Brucella abortus* (BtpB, UniProt ID Q2YN91) (Salcedo et al., 2013), *Salmonella enterica* serovar Enteritidis (TlpA, UniProt ID A0A3V4TC50) (Newman et al., 2006), *Paracoccus denitrificans* (PdTLP, UniProt ID A1AY86) (Chan et al., 2009; Low et al., 2007), *Staphylococcus aureus* (TirS and SaTlp1, UniProt ID M1XK12 and D2N983, respectively) (Askarian et al., 2014; Patterson et al., 2014), *Yersinia pestis* (YpTdp, UniProt ID Q8CL16) (Rana et al., 2011), *Helicobacter pylori* (HP1437, UniProt ID O25978) (Kaplan-Türköz, 2017), *Yersinia pseudotuberculosis* serotype O:1b (TcpYI, UniProt ID A0A0U1QVR5) (Nörenberg et al., 2013), and *Yersinia pseudotuberculosis* serotype O:3 (TcpYIII, UniProt ID A0A0H3B787) (Nörenberg et al., 2013). The sequences of the above-mentioned proteins were used for the analysis and construction of the three-dimensional (3D) models of their NTDs using computational modeling approaches. The primary structures of the NTDs were analyzed using the ExPASy-ProtParam tool (Gasteiger et al., 2005). The NTDs boundaries involve the amino acids along the N-terminus prior to the TIR domain of the selected bacterial Tcps (Fig. 1).

### Physicochemical characterization

Physicochemical properties including the molecular weight (Mwt), amino acid composition (AA), theoretical isoelectric point (pI), number of negative residues (-R), number of positive residues (+R), extinction coefficient at 280 nm (EC), half-life (hrs), instability index (II), aliphatic index (AI) and grand average of hydropathy (GRAVY) of the NTDs of selected bacterial Tcps were analyzed using ExPASy-ProtParam tool (Gasteiger et al., 2005).

### Secondary structure prediction

For the prediction of secondary structure components, two web servers were used, including SOPMA (Geourjon & Deléage, 1995) and GOR IV (Garnier, Gibrat & Robson, 1996). The default parameters were used. The percentages of secondary structure components were predicted based on the analysis of relative frequencies of each amino acid in the helices, sheets and turns present in the X-ray crystallographic templates of the proteins.

### Multiple sequence alignment

Multiple sequence alignment for the NTDs of the selected bacterial Tcps was performed using the Clustal Omega server (Madeira et al., 2019) and viewed using the Jalview server (Waterhouse et al., 2009).

### Construction and evaluation of protein models

The 3D models of the NTDs of the selected bacterial Tcps were constructed using three homology modeling servers, including Phyre2 (Kelley et al., 2015), SWISS-MODEL (Waterhouse et al., 2018) and I-TASSER (Yang & Zhang, 2015). The available default and/or automated options were used. After optimization, the 3D models were verified using the RAMPAGE (DePristo, De Bakker & Blundell, 2004; Lovell et al., 2003) and ProSA-web (Sippl, 1993; Wiederstein & Sippl, 2007) servers. RAMPAGE validates 3D models by plotting the Ramachandran plot. Generally, the best models exhibit high percentage of the total number of residues in the most favored regions and additional allowed regions and less percentage of the residues in the disallowed or outlier regions of the Ramachandran plot. ProSA-web server validates the quality of the protein models using available protein structures derived from PDB based on z-scoring system. Models were visualized using PyMOL (The PyMOL molecular graphics system, Version 2.0 Schrödinger, LLC).

## Results and Discussion

### Prediction and characterization of primary protein sequences of NTDs of the selected bacterial Tcps

The amino acid sequences of the NTDs of the selected bacterial Tcps were retrieved from UniProt (http:/www.uniprot.org). The details of the unique UniProt IDs, amino acid sequence boundaries and bacterial species of the NTDs of the selected proteins are provided in Table 1. We use the ExPASy-ProtParam tool (Gasteiger et al., 2005) to analyze the proteins primary structures and compute different parameters for their physicochemical properties (Table 2 and Table 3). All 20 amino acids were detected, of which the percentage of alanine, isoleucine, leucine, lysine and serine was the highest, while that of tryptophan and cysteine was the lowest (Table 2).

**Table 1:** Amino acid boundaries, UniProt IDs, and bacterial species of NTDs of the selected bacterial Tcps.

**NTD protein name**	**Amino acid boundaries**	**UniProt ID**	**Organism**
N-TcpC	1-170	A0A0H2V8B5	Uropathogenic *Escherichia coli* O6:H1 CFT073
N-TIpA	1-159	A0A3V4TC50	*Salmonella enterica* serovar Enteritidis CFSAN000052
N-BtpA	1-118	Q8YF53	*Brucella melitensis* biotype 1 16M
N-BtpB	1-156	Q2YN91	*Brucella abortus* 2308
N-PdTLP	1-165	A1AY86	*Paracoccus denitrificans* 1222
N-YpTdp	1-136	Q8CL16	*Yersinia pestis*
N-TirS	1-142	M1XK12	*Staphylococcus aureus*
N-SaTIp1	1-202	D2N983	*Staphylococcus aureus* ST398
N-HP1437	1-83	O25978	*Helicobacter pylori* 26695
N-TcpYI	1-92	A0A0U1QVR5	*Yersinia pseudotuberculosis* serotype O:1b IP 31758
N-TcpYIII	1-91	A0A0H3B787	*Yersinia pseudotuberculosis* serotype O:3 YPIII

**Notes.**

Amino acid boundaries are defined by the position number of the amino acid in the primary four sequences of the full-length bacterial Tcps.

**Table 2:** Number and percentages of amino acids present in the NTDs of selected bacterial Tcps estimated by ExPASy-ProtParam tool.

**Amino acid**	**No. (%)**										
	**N-TcpC**	**N-TIpA**	**N-BtpA**	**N-BtpB**	**N-PdTLP**	**N-YpTdp**	**N-TirS**	**N-SaTIp1**	**N-HP1437**	**N-TcpYI**	**N-TcpYIII**
**A (Ala)**	11 (6.5)	7 (4.4)	20 (16.9)	12 (7.7)	34 (20.6)	6 (4.4)	4 (2.8)	11 (5.4)	6 (7.2)	10 (10.9)	10 (11.0)
**R (Arg)**	4 (2.4)	10 (6.3)	6 (5.1)	9 (5.8)	12 (7.3)	4 (2.9)	8 (5.6)	11 (5.4)	1 (1.2)	8 (8.7)	9 (9.9)
**N (Asn)**	21 (12.4)	10 (6.3)	0 (0)	5 (3.2)	5 (3.0)	9 (6.6)	14 (9.9)	15 (7.4)	9 (10.8)	5 (5.4)	3 (3.3)
**D (Asp)**	7 (4.1)	9 (5.7)	5 (4.2)	11 (7.1)	6 (3.6)	9 (6.6)	6 (4.2)	8 (4.0)	6 (7.2)	0 (0)	0 (0)
**C (Cys)**	1 (0.6)	1 (0.6)	0 (0)	3 (1.9)	0 (0)	2 (1.5)	0 (0)	0 (0)	0 (0)	3 (3.3)	3 (3.3)
**Q (Gln)**	7 (4.1)	14 (8.8)	12 (10.2)	7 (4.5)	11 (6.7)	5 (3.7)	11 (7.7)	10 (5.0)	4 (4.8)	3 (3.3)	0 (0)
**E (Glu)**	10 (5.9)	11 (6.9)	11 (9.3)	12 (7.7)	13 (7.9)	14 (10.3)	14 (9.9)	18 (8.9)	4 (4.8)	1 (1.1)	3 (3.3)
**G (Gly)**	4 (2.4)	2 (1.3)	2 (1.7)	8 (5.1)	4 (2.4)	3 (2.2)	1 (0.7)	3 (1.5)	1 (1.2)	9 (9.8)	12 (13.2)
**H (His)**	4 (2.4)	3 (1.9)	2 (1.7)	4 (2.6)	0 (0)	6 (4.4)	0 (0)	4 (2.0)	2 (2.4)	1 (1.1)	1 (1.1)
**I (Ile)**	22 (12.9)	16 (10.1)	5 (4.2)	8 (5.1)	6 (3.6)	16 (11.8)	14 (9.9)	13 (6.4)	7 (8.4)	1 (1.1)	0 (0)
**L (Lue)**	15 (8.8)	11 (6.9)	5 (4.2)	14 (9.0)	16 (9.7)	13 (9.6)	13 (9.2)	15 (7.4)	5 (6.0)	1 (1.1)	1 (1.1)
**K (Lys)**	10 (5.9)	25 (15.7)	20 (16.9)	9 (5.8)	21 (12.7)	7 (5.1)	26 (18.3)	40 (19.8)	13 (15.7)	1 (1.1)	2 (2.2)
**M (Met)**	3 (1.8)	3 (1.9)	4 (3.4)	2 (1.3)	4 (2.4)	2 (1.5)	5 (3.5)	4 (2.0)	2 (2.4)	2 (2.2)	1 (1.1)
**F (Phe)**	15 (8.8)	1 (0.6)	2 (1.7)	9 (5.8)	0 (0)	2 (1.5)	0 (0)	7 (3.5)	4 (4.8)	0 (0)	0 (0)
**P (Pro)**	3 (1.8)	2 (1.3)	0 (0)	7 (4.5)	5 (3.0)	8 (5.9)	2 (1.4)	3 (1.5)	4 (4.8)	4 (4.3)	8 (8.8)
**S (Ser)**	10 (5.9)	20 (12.6)	15 (12.7)	4 (2.6)	12 (7.3)	8 (5.9)	12 (8.5)	7 (3.5)	9 (10.8)	26 (28.3)	19 (20.9)
**T (Thr)**	4 (2.4)	10 (6.3)	6 (5.1)	12 (7.7)	12 (7.3)	3 (2.2)	7 (4.9)	10 (5.0)	1 (1.2)	6 (6.5)	7 (7.7)
**W (Trp)**	4 (2.4)	0 (0)	0 (0)	5 (3.2)	0 (0)	2 (1.5)	0 (0)	1 (0.5)	0 (0)	1 (1.1)	1 (1.1)
**Y (Tyr)**	11 (6.5)	2 (1.3)	1 (0.8)	4 (2.6)	1 (0.6)	7 (5.1)	1 (0.7)	15 (7.4)	2 (2.4)	6 (6.5)	6 (6.6)
**V (Val)**	4 (2.4)	2 (1.3)	2 (1.7)	11 (7.1)	3 (1.8)	10 (7.4)	4 (2.8)	7 (3.5)	3 (3.6)	4 (4.3)	5 (5.5)

**Table 3:** Physicochemical properties of NTD proteins of selected bacterial Tcps.

**Protein name**	**AA**	**Mwt**	**pI**	**-R(Asp + Glu)**	**+R(Arg + Lys)**	**EC (assuming all Cys residues from cystines)**	**EC (assuming all Cys residues are reduced)**	**Half-life (hrs)**	**II**	**AI**	**GRAVY**
**N-TcpC**	170	20.27	5.77	17	14	38390	38390	30	45.12	98.18	−0.115
**N-TIpA**	159	18.37	9.97	20	35	2980	2980	30	60.04	74.28	−1.194
**N-BtpA**	118	13.09	9.87	16	26	1490	1490	30	51.09	54.92	−1.090
**N-BtpB**	156	18.02	5.37	23	18	33585	33460	30	24.77	83.14	−0.340
**N-PdTLP**	165	17.80	10.15	19	33	1490	1490	30	38.62	77.88	−0.716
**N-YpTdp**	136	15.80	4.74	23	11	21555	21430	30	48.17	108.9	−0.285
**N-TirS**	142	16.66	9.96	20	34	1490	1490	30	53.88	85.14	−1.186
**N-SaTIp1**	202	24.57	9.89	26	51	27850	27850	30	44.08	69.55	−1.207
**N-HP1437**	83	9.45	9.40	10	14	2980	2980	30	39.15	74.10	−0.849
**N-TcpYI**	92	9.46	10.15	1	9	14565	14440	30	79.08	31.96	−0.695
**N-TcpYIII**	91	9.30	10.03	3	11	14565	14440	30	63.47	31.21	−0.733

**Notes.**

The table shows, for each NTD protein, the number of amino acids (AA), molecular weight (Mwt), isoelectric point (pI), number of negative residues (-R), number of positive residues (+R), extinction coefficient at 280 nm (EC), instability index (II), aliphatic index (AI), and grand average of hydropathicity (GRAVY).

In this study, the molecular weight (Mwt) of NTDs varied from 9.30 kDa (*Yersinia pseudotuberculosis* NTD) to 24.57 kDa (*Staphylococcus aureus* NTD) (Table 3). ExPASy-ProtParam tool computes the extinction coefficient (EC) at wavelength 280 nm. Table 3 shows several NTDs with high EC values at 280 nm, including N-TcpC, N-BtpB, N-SaTLP1 and N-YpTdp (EC values of 38390, 33585, 27850 and 21555, respectively), with respect to the concentrations of cystine, Trp and Tyr (cysteine does not absorb light appreciably at wavelengths >260 nm, while cystine does (Gasteiger et al., 2005) (see Table 2). Knowing the EC value of a protein might help scientists in optimizing the purification procedure of their protein of interest (Gasteiger et al., 2005). The instability index (II) values of the NTDs of the selected proteins are shown in Table 3. The II provides an estimate of the stability of the protein of interest in a test tube. If the II of a protein is below 40, then the protein is considered to be stable, and if the II is above 40, then the protein is considered to be unstable (Gasteiger et al., 2005). Therefore, the results (Table 3) show that NTDs from PdTLP, BtpB and HP1437 are predicted to be stable in a test tube.

The isoelectric point (pI) is the pH of the solution at which the net charge of the surface amino acids of a protein equal to zero (Gasteiger et al., 2005). The computed pI values of the NTDs are shown in Table 3. The pI of the protein varies from acidic, as for N-YpTdp (pI = 4.74), to alkaline, as for N-PdTLP (pI = 10.15) and N-TcpYI (pI = 10.15). The computed pI value is useful for screening a suitable buffering system for the purification of the protein of interest, which is important for ensuring the stability of the purified protein (Gasteiger et al., 2005). In addition, Table 3 shows the total number of negatively charged residues (-R (Asp + Glu)) and the total number of positively charged residues (+R (Arg + Lys)). All NTDs consisted of fewer negatively charged residues than positively charged residues except for N-TcpC, N-YpTdp and N-BtpB. Negatively charged amino acid residues (i.e., Ala, Asp and Glu) are polar and hydrophilic in nature, and they are accessible to the surrounding environment as parts of proteins. When a protein has fewer negatively charged residues than positively charged residues, it may reflect that the protein is involved in intercellular protein-protein interactions (Bhagavan & Ha, 2011). The aliphatic index (AI) of a protein is defined as the relative volume occupied by aliphatic side chains, which include Ala, Val, Ile and Leu, and contribute to protein thermostability (Gasteiger et al., 2005). The AIs for the NTDs of selected proteins are shown in Table 3. High AI means that proteins are predicted to be thermally stable and are hydrophobic in nature (i.e., they contain a large number of hydrophobic amino acids, including Met, Ala, Leu, Gly, Pro, Phe, Ile, Val and Trp) (Gurskaia, 1968). Table 3 shows that, in this study, the majority of NTDs had high AI except for N-BtpA, N-TcpYI and N-TcpYIII, indicating their low thermal stability.

The grand average of hydropathy (GRAVY) for a peptide or protein is the summision of hydropathy values of all amino acids divided by the number amino acids in the sequence (Gasteiger et al., 2005). When a protein is found to have greater negative GRAVY value, this reflects the hydrophilic nature of the protein and the possibility of better interaction between the protein and water (Gasteiger et al., 2005). In this study, NTDs, including N-TlpA, N-BtpA, N-TirS and N-SaTlp1, were found to be more hydrophilic compared to other NTDs (Table 3). ExpPASy-ProtParam tool is used to predict the half-life of proteins. It can predict the time it takes for half of the amount of protein in a cell to degrade after the protein has been synthesized (Bojkowska et al., 2011). The ExpPASy-ProtParam tool can predict the half-life of proteins of three organisms, including human, *Escherichia coli*, and yeast. However, the tool can be used to estimate the half-life of similar organisms (Gasteiger et al., 2005). In our study, the half-life of all NTDs was found to be similar (equal to 30 hrs), suggesting that the NTDs have long half-life. According to Moran et al. (2013), a typical bacterial protein has half-life of 20 hrs. Therefore, the half-life of the NTDs in this study needs further investigation both in vivo and/or in vitro (Bojkowska et al., 2011; Reder, Michalik & Gerth, 2018).

**Table 4:** Prediction of the percentages of secondary structure components in NTDs of selected bacterial Tcps using SOPMA and GOR IV servers.

	**N-TcpC**	**N-TIpA**	**N-BtpA**	**N-BtpB**	**N-PdTLP**	**N-YpTdp**	**N-TirS**	**N-SaTIp1**	**N-HP1437**	**N-TcpYI**	**N-TcpYIII**	
**Alpha helix**	41.18	88.05	100	38.46	76.97	41.91	85.92	58.42	46.99	21.74	14.29	**SOPMA**
	39.41	52.83	90.68	30.13	85.45	47.06	73.94	59.41	44.58	7.61	0	**GOR IV**
**Betasheet**	20	3.14	0	25	7.28	19.86	2.81	15.85	20.48	7.6	14.29	**SOPMA**
	18.82	8.81	1.69	17.95	3.03	9.56	2.11	3.96	7.23	9.78	14.29	**GOR IV**
**Random coil**	28.82	8.81	0	36.54	15.76	38.24	11.27	25.74	32.53	70.65	71.43	**SOPMA**
	41.76	38.36	7.63	51.92	11.52	43.38	23.94	36.63	48.19	82.61	85.71	**GOR IV**

### Prediction and characterization of the secondary structures of NTDs of the selected bacterial Tcps

The secondary structures of the NTDs of the selected bacterial Tcps were predicted using SOPMA (Geourjon & Deléage, 1995) and GOR IV (Kouza et al., 2017) servers. Both tools showed the presence of various percentages of the secondary structure components between the NTDs, including alpha helices, beta sheets and random coils (Table 4). This could be explained by the low sequence similarity and identity between NTDs of the selected bacterial Tcps (Fig. 1). The predicted secondary structure components show that N-TIpA, N-BtpA, N-PdTLP, N-TirS and N-SaTIp1 contain high percentages of alpha helices. Previous studies reported that coiled-coil structure of the NTDs from BtpA and TlpA are suggested to facilitate Tcps dimerization and/or colocalization into the plasma membrane (Alaidarous et al., 2014; Xiong et al., 2019). This allows the bacterial Tcps to mimic the function of adaptor proteins by binding to TLRs resulting in the suppression of the host proinflammatory response (Radhakrishnan et al., 2009; Rana et al., 2013; Ve et al., 2012). In addition, we suggest that the bacterial Tcps including N-TcpC, N-BtpB, N-YpTdp, N-HP1437, N-TcpYI and N-TcpYIII are likely to have their NTDs involved in the interaction with the adaptor proteins, as their secondary structure prediction showed high percentages of coiled turns. The protein-protein interaction between bacterial Tcps and the host adaptor proteins such as the myeloid differentiation factor 88 (MyD88) and the MyD88 adaptor-like (MAL) proteins is another suggested molecular function of bacterial Tcps (Ve, Williams & Kobe, 2015). However, there is no evidence of a direct interaction between these proteins reported yet. The variations in the secondary structure components between the NTDs of the selected bacterial Tcps suggest that these proteins might have different strategies for suppressing the TLR signaling pathways. However, the exact molecular mechanism of bacterial Tcp-dependent immune suppression is still unclear.

### Three-dimensional structural modeling and validation of the NTDs of the selected bacterial Tcps

Although the structures of TIR domains of several bacterial Tcps have been determined, the structures of full-length proteins or other domains in the Tcps have not yet been determined (Alaidarous et al., 2014; Chan et al., 2009; Kaplan-Türköz et al., 2013). Here, we have constructed models of the NTDs of several bacterial Tcps. The 3D models of the NTDs were constructed using three homology modeling servers, including Phyre2 (Kelley et al., 2015), SWISS-MODEL (Waterhouse et al., 2018) and I-TASSER (Roy, Kucukural & Zhang, 2010). The three modeling servers produced similar NTD models for individual NTD protein (Table 5). For example, the servers predicted coiled-coil structures containing high alpha helices for N-TIpA, N-BtpA, N-PdTLP, N-TirS and N-SaTIp1 (Table 5). Interestingly, this is consistent with the secondary structure prediction for these proteins where these proteins are predicted to have more than 50% alpha helices contents (Table 4). In addition, the secondary structure prediction of N-TcpC, N-BtpB, N-YpTdp, N-HP1437, N-TcpYI and N-TcpYIII (Table 4) are consistent with the models generated for the proteins, containing low alpha helices and more of other secondary structural components (Table 5). As part of the virulence mechanisms, studies have suggested that dimerization of bacterial Tcps are required for the binding to signaling and/or adaptor proteins or binding to the phosphoinositides in the plasma membrane (Alaidarous et al., 2014; Fekonja, Benčina & Jerala, 2012; Radhakrishnan et al., 2009; Rana et al., 2013; Ve, Williams & Kobe, 2015). Therefore, based on the structural prediction in this study, it is suggested that bacterial Tcps with the predicted coiled-coil NTD structures (including N-TIpA, N-BtpA, N-PdTLP, N-TirS and N-SaTIp1) are likely to be involved in bacterial Tcps dimerization facilitating the binding to signaling and/or adaptor proteins or binding to the phosphoinositides in the plasma membrane. In addition, NTDs proteins with the prediction of having low alpha helices contents (including N-TcpC, N-BtpB, N-YpTdp, N-HP1437, N-TcpYI and N-TcpYIII) are likely to be involved only in the protein-protein interaction. Future biochemical and experimental structural studies will bring insights into the exact molecular mechanism behind the bacterial Tcps-dependent signaling inhibition (Elde & Malik, 2009; Patterson et al., 2014).

**Table 5:** 3D models of the NTD proteins of selected bacterial Tcps using three modeling servers; Phyre2, SWISS-MODEL and I-TASSER.

	**Modeling server**		
**NTD protein (amino acid boundary)**	**Phyre2**	**SWISS-MODEL**	**I-TASSER**
**N-TcpC(1-170)**	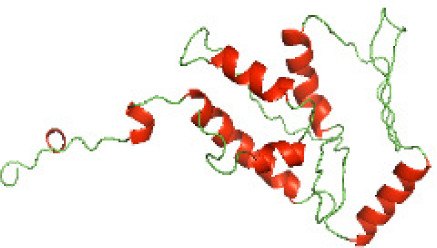	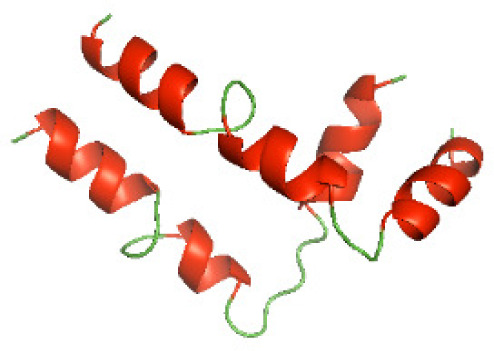	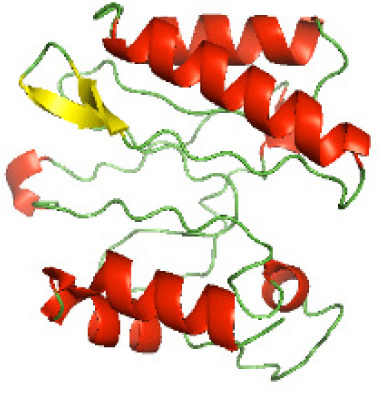
**N-TIpA(1-159)**	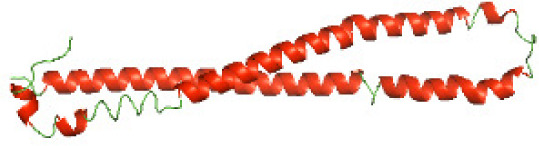	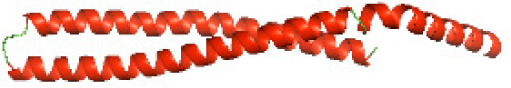	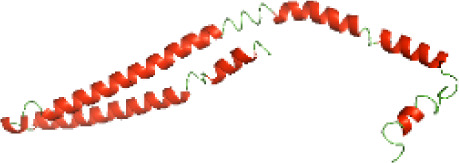
**N-BtpA(1-118)**	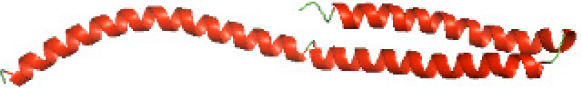	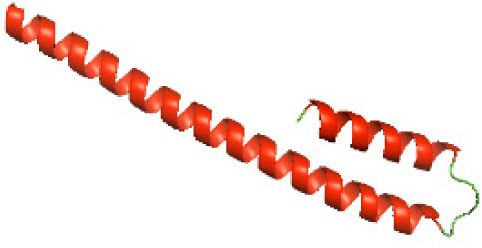	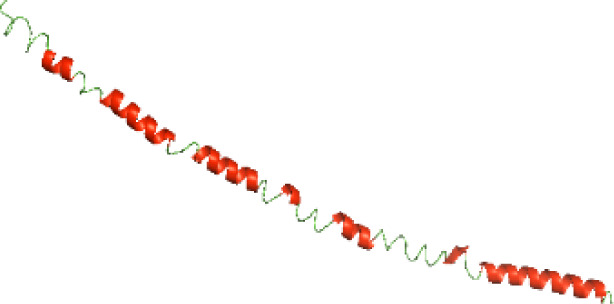
**N-BtpB(1-156)**	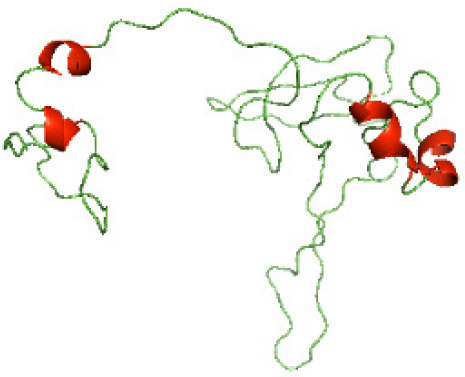	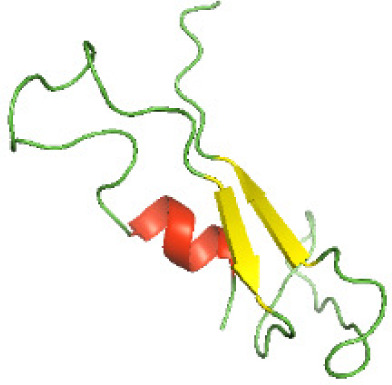	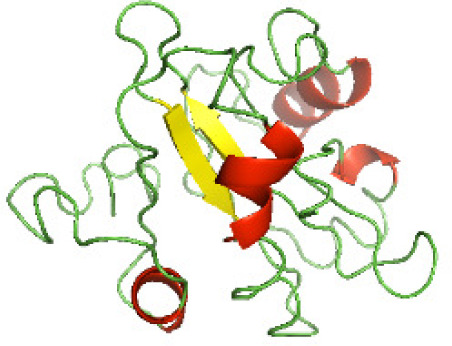
**N-PdTLP(1-165)**	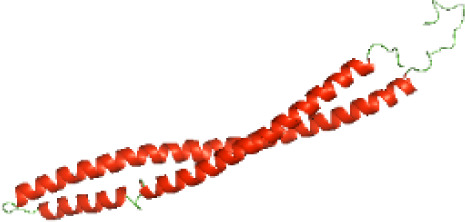	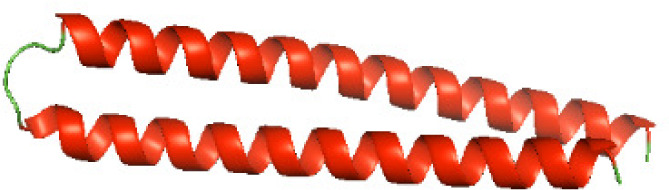	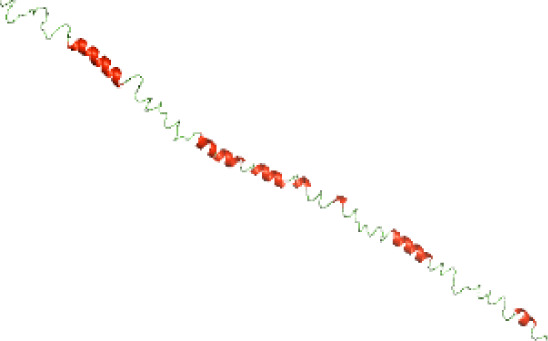
**N-YpTdp(1-136)**	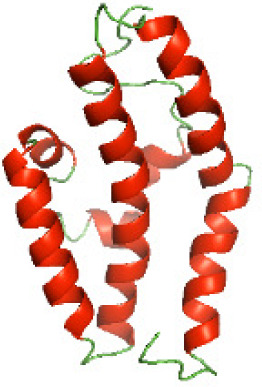	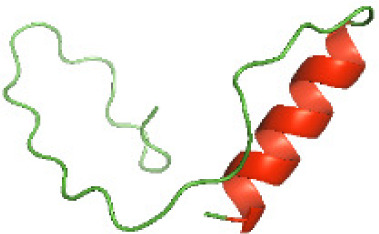	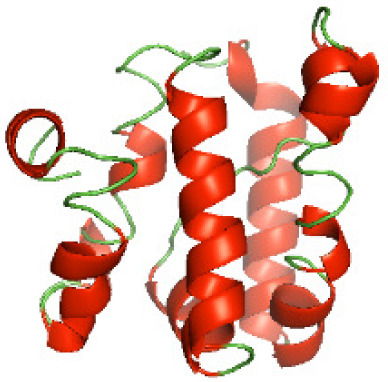
**N-TirS(1-142)**	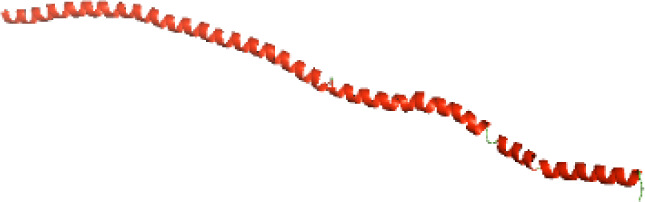	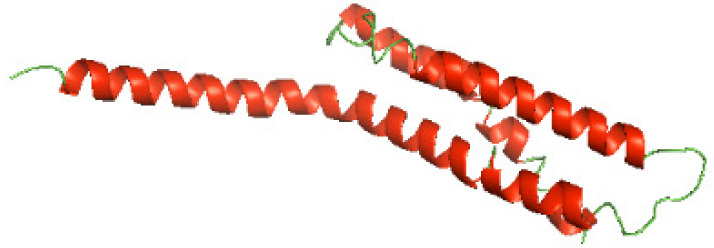	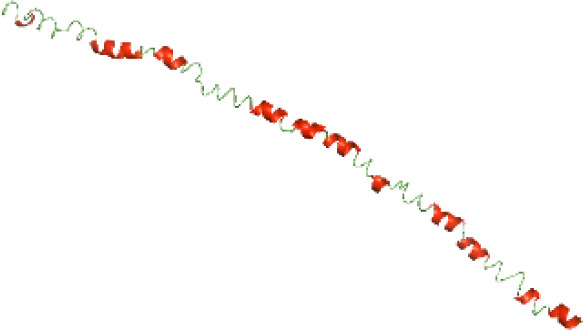
**N-SaTIp1(1-202)**	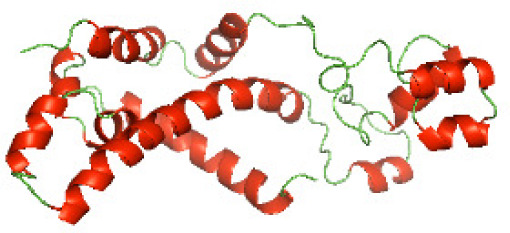	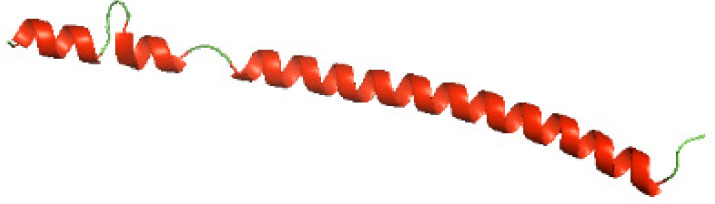	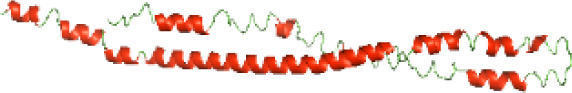
**N-HP1437(1-83)**	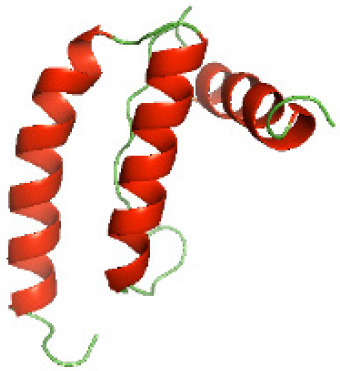	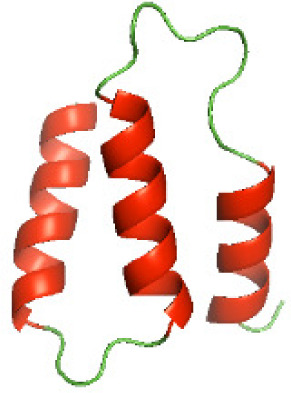	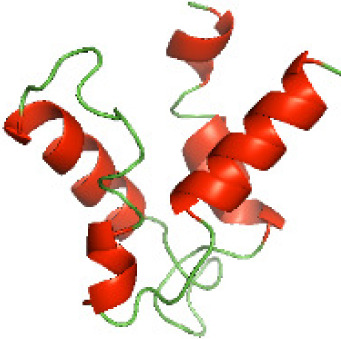
**N-TcpYI(1-92)**	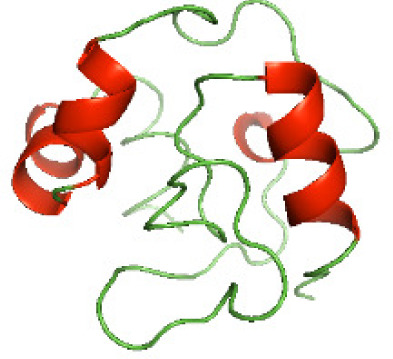	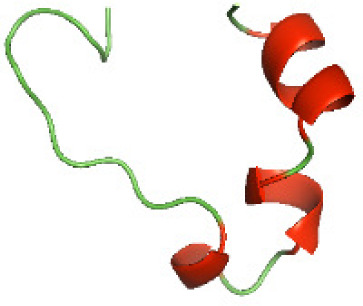	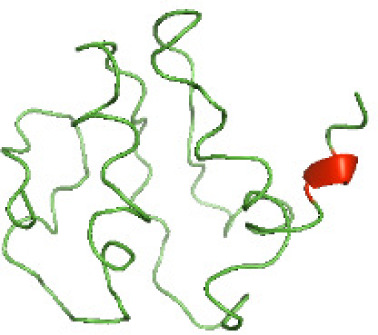
**N-TcpYIII(1-91)**	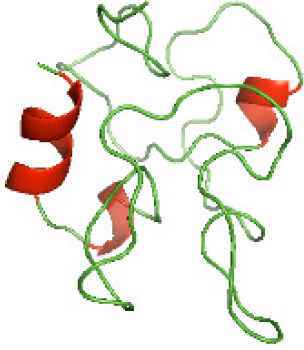	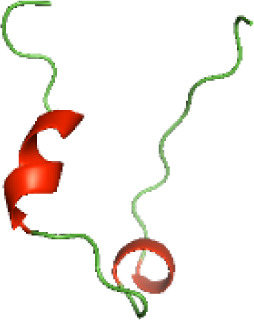	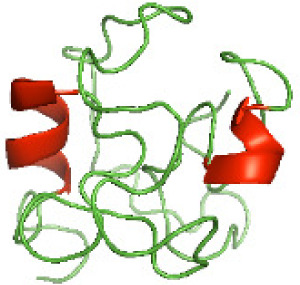

**Table 6:** Ramachandran plot calculation using RAMPAGE server.

**Server**	**Ramachandran plot calculation(% residues)**	**N-TcpC**	**N-TIpA**	**N-BtpA**	**N-BtpB**	**N-PdTLP**	**N-YpTdp**	**N-TirS**	**N-SaTIp1**	**N-HP1437**	**N-TcpYI**	**N-TcpYIII**
**Phyre2**	Favored region	73.2	87.3	96.6	64.3	96.3	79.1	95.7	82.5	86.4	62.2	49.4
	Allowed region	14.9	7.6	1.7	14.3	3.1	12.7	2.9	9.0	9.9	20	18.0
	Disallowed region	11.9	5.1	1.7	21.4	0.6	8.2	1.4	8.5	3.7	17.8	32.6
**SWISS-MODEL**	Favored region	89.7	100	94.2	82.5	95.2	83.7	91.5	93.8	91.2	96.4	85.7
	Allowed region	7.4	0	2.9	14.3	2.4	16.3	7.0	3.1	5.3	3.6	14.3
	Disallowed region	2.9	0	2.9	3.2	2.4	0	1.6	3.1	3.5	0	0
**I-TASSER**	Favored region	68.5	77.7	72.4	55.8	65.0	86.6	69.3	69.0	65.4	40.0	50.6
	Allowed region	19	12.1	23.3	24.0	23.3	6.7	21.4	17.5	23.5	34.4	27.0
	Disallowed region	12.5	10.2	4.3	20.1	11.7	6.7	9.3	13.5	11.1	25.6	22.5

**Table 7:** *Z*-scores for overall model quality calculated using the ProSA-web server.

		**Server**			
		**Phyre2**	**SWISS-MODEL**	**I-TASSER**	
**NTD protein**	**N-TcpC**	−1.97	−0.91	−3.19	***z*-score**
	**N-TIpA**	−3.12	−2.88	−2.84	
	**N-BtpA**	−2.35	−2.51	−1.85	
	**N-BtpB**	−1.43	−1.73	−3.17	
	**N-PdTLP**	−3.77	−3.39	−1.66	
	**N-YpTdp**	−3.16	−0.94	−5.10	
	**N-TirS**	−1.70	−1.88	−0.94	
	**N-SaTIp1**	−2.72	−0.49	−2.00	
	**N-HP1437**	−3.22	−2.93	−5.52	
	**N-TcpYI**	−1.30	−0.24	−2.40	
	**N-TcpYIII**	−0.97	−0.47	−2.94	

RAMPAGE software used in this study to validate the constructed 3D models of the proteins. RAMPAGE uses the Ramachandran plot, which presents the backbone conformation of proteins based on the position of non-Gly residues in the disallowed regions and phi/psi distribution in the model. The RAMPAGE score is an estimation of the absolute quality of a model. Determined by comparing the model to similar-sized reference experimentally solved structures present in the Protein Data Bank (PDB) (Lovell et al., 2003). The models of the NTDs were analyzed by RAMPAGE, which revealed that the majority of the residues fell in the favored and allowed regions of the Ramachandran plot (Table 6). This indicated the good quality of the models constructed using the three modeling servers. N-BtpB, N-TcpYI, and N-TcpYIII showed high percentage of outliers for all models except the one generated using the SWISS-MODEL server.

The modeled structures of the NTDs were also validated using the ProSA-web server (Wiederstein & Sippl, 2007). ProSA-web gives a score that indicates the “degree of nativeness” of the modeled protein structures, called as z-score. The value of z-score indicates the quality of the modeled protein structure, with large negative z-score indicating native fold while scores closer to positive values indicating problematic or erroneous models (Wiederstein & Sippl, 2007). In this study, the z-scores for all the three models of N-TlpA, N-BtpA, N-BtpB, N-PdTLP, and N-HP1437 were found to be highly negative (Table 7). This indicates that the models are of reasonable quality and exhibit high degree of native fold. Although in case of other NTDs, the z-scores of each of the three models are different (Table 7), further experimental studies are required to determine the consistency of these models. In addition, the servers available for constructing protein models use available protein sequence data and known protein structures to generate protein structural models (Kelley et al., 2015; Roy, Kucukural & Zhang, 2010; Waterhouse et al., 2018). This suggests that scientists need to perform a greater number of structural studies on bacterial Tcps in order to improve the computational structural modeling tools. Aiming towards producing quality protein models highlighting the mechanism behind the bacterial Tcps molecular function.

## Conclusions

Understanding the molecular mechanism of bacterial Tcps-dependent evasion strategy employed to suppress the host immune response using experimental approaches is challenging. Most studies have reported low protein solubility when the bacterial Tcp is expressed at full-length or as protein domains (Patterson et al., 2014; Rana et al., 2013; Salcedo et al., 2013; Ve et al., 2012). *In silico* homology modeling studies provide an opportunity to establish a pipeline for structural modeling and analysis of any protein as part of understanding the molecular mechanism of the protein function and determining therapeutic targets (Ke et al., 2016; Sliwoski et al., 2014; Ve et al., 2012). In this study, NTDs of selected bacterial Tcps were selected for physicochemical characterization and homology structural modeling using in silico approaches. The study presents the physicochemical characteristics of selected NTDs that are vital for protein stability during the purification of the proteins. In addition, the study provides the characteristics of secondary structures and 3D models (of acceptable quality) of the NTDs. This study will be the base of future biochemical and experimental structural studies, which may aid in elucidating the functional molecular mechanism of the pathogenic bacterial Tcps.

## Additional Information and Declarations

### Data Availability

The following information was supplied regarding data availability: Sequence data are available at UniProt: A0A0H2V8B5 (TcpC), A0A3V4TC50 (TIpA), Q8YF53 (BtpA), Q2YN91 (BtpB), A1AY86 (PdTLP), Q8CL16 (YpTdp), M1XK12 (TirS), D2N983 (SaTIp1), O25978 (HP1437), A0A0U1QVR5 (TcpYI), A0A0H3B787 (TcpYIII).

## References

[ref-1] Alaidarous M, Ve T, Casey LW, Valkov E, Ericsson DJ, Ullah MO, Schembri MA, Mansell A, Sweet MJ, Kobe B (2014). Mechanism of bacterial interference with TLR4 signaling by Brucella Toll/interleukin-1 receptor domain-containing protein TcpB. Journal of Biological Chemistry.

[ref-2] Askarian F, vanSorge NM, Sangvik M, Beasley FC, Henriksen JR, Sollid JU, vanStrijp JA, Nizet V, Johannessen M (2014). A Staphylococcus aureus TIR domain protein virulence factor blocks TLR2-mediated NF-κB signaling. Journal of Innate Immunity.

[ref-3] Bhagavan NV, Ha C-E (2011). Essentials of medical biochemistry: with clinical cases.

[ref-4] Bojkowska K, SantonideSio F, Barde I, Offner S, Verp S, Heinis C, Johnsson K, Trono D (2011). Measuring in vivo protein half-life. Chemistry and Biology.

[ref-5] Chan SL, Low LY, Hsu S, Li S, Liu T, Santelli E, Negrate GLe, Reed JC, Woods VL, Pascual J (2009). Molecular mimicry in innate immunity: crystal structure of a bacterial TIR domain. Journal of Biological Chemistry.

[ref-6] DePristo MA, De Bakker PI, Blundell TL (2004). Heterogeneity and inaccuracy in protein structures solved by X-ray crystallography. Structure.

[ref-7] Elde NC, Malik HS (2009). The evolutionary conundrum of pathogen mimicry. Nature Reviews. Microbiology.

[ref-8] Fekonja O, Benčina M, Jerala R (2012). Toll/interleukin-1 receptor domain dimers as the platform for activation and enhanced inhibition of Toll-like receptor signaling. Journal of Biological Chemistry.

[ref-9] Garnier J, Gibrat JF, Robson B (1996). GOR method for predicting protein secondary structure from amino acid sequence. Methods in Enzymology.

[ref-10] Gasteiger E, Hoogland C, Gattiker A, DuvaudSe, Wilkins MR, Appel RD, Bairoch A, Walker JM (2005). Protein Identification and Analysis Tools on the ExPASy Server. The Proteomics Protocols Handbook.

[ref-11] Geourjon C, Deléage G (1995). SOPMA: significant improvements in protein secondary structure prediction by consensus prediction from multiple alignments. Computer Applications in the Biosciences.

[ref-12] Gurskaia GV (1968). The molecular structure of amino acids; determination by X-ray diffraction analysis.

[ref-13] Imbert PR, Louche A, Luizet JB, Grandjean T, Bigot S, Wood TE, Gagné S, Blanco A, Wunderley L, Terradot L, Woodman P, Garvis S, Filloux A, Guery B, Salcedo SP (2017). A Pseudomonas aeruginosa TIR effector mediates immune evasion by targeting UBAP1 and TLR adaptors. EMBO Journal.

[ref-14] Kaplan-Türköz B (2017). A putative Toll/interleukin-1 receptor domain protein from Helicobacter pylori is dimeric in solution and interacts with human Toll-like receptor adaptor myeloid differentiation primary response 88. Microbiology and Immunology.

[ref-15] Kaplan-Türköz B, Koelblen T, Felix C, Candusso MP, O’Callaghan D, Vergunst AC, Terradot L (2013). Structure of the Toll/interleukin 1 receptor (TIR) domain of the immunosuppressive Brucella effector BtpA/Btp1/TcpB. FEBS Letters.

[ref-16] Kawai T, Akira S (2010). The role of pattern-recognition receptors in innate immunity: update on Toll-like receptors. Nature Immunology.

[ref-17] Ke Y, Li W, Wang Y, Yang M, Guo J, Zhan S, Du X, Wang Z, Li J, Chen Z (2016). Inhibition of TLR4 signaling by Brucella TIR-containing protein TcpB-derived decoy peptides. International Journal of Medical Microbiology.

[ref-18] Kelley LA, Mezulis S, Yates CM, Wass MN, Sternberg MJ (2015). The Phyre2 web portal for protein modeling, prediction and analysis. Nature Protocols.

[ref-19] Kouza M, Faraggi E, Kolinski A, Kloczkowski A (2017). The GOR Method of Protein Secondary Structure Prediction and Its Application as a Protein Aggregation Prediction Tool. Methods in Molecular Biology.

[ref-20] Kraemer TD, QuintanarHaro OD, Domann E, Chakraborty T, Tchatalbachev S (2014). The TIR domain containing locus of enterococcus faecalis is predominant among urinary tract infection isolates and downregulates host inflammatory response. International Journal of Microbiology.

[ref-21] Lovell SC, Davis IW, Arendall WB, De Bakker PI, Word JM, Prisant MG, Richardson JS, Richardson DC (2003). Structure validation by Calpha geometry: phi, psi and Cbeta deviation. Proteins.

[ref-22] Low LY, Mukasa T, Reed JC, Pascual J (2007). Characterization of a TIR-like protein from Paracoccus denitrificans. Biochemical and Biophysical Research Communications.

[ref-23] Madeira F, Park YM, Lee J, Buso N, Gur T, Madhusoodanan N, Basutkar P, Tivey ARN, Potter SC, Finn RD, Lopez R (2019). The EMBL-EBI search and sequence analysis tools APIs in 2019. Nucleic Acids Research.

[ref-24] Moran MA, Satinsky B, Gifford SM, Luo H, Rivers A, Chan LK, Meng J, Durham BP, Shen C, Varaljay VA, Smith CB, Yager PL, Hopkinson BM (2013). Sizing up metatranscriptomics. ISME Journal.

[ref-25] Newman RM, Salunkhe P, Godzik A, Reed JC (2006). Identification and characterization of a novel bacterial virulence factor that shares homology with mammalian Toll/interleukin-1 receptor family proteins. Infection and Immunity.

[ref-26] Nörenberg D, Wieser A, Magistro G, Hoffmann C, Meyer C, Messerer M, Schubert S (2013). Molecular analysis of a novel Toll/interleukin-1 receptor (TIR)-domain containing virulence protein of Y. pseudotuberculosis among Far East scarlet-like fever serotype I strains. International Journal of Medical Microbiology.

[ref-27] Patterson NJ, Günther J, Gibson AJ, Offord V, Coffey TJ, Splitter G, Monk I, Seyfert HM, Werling D (2014). Two TIR-like domain containing proteins in a newly emerging zoonotic Staphylococcus aureus strain sequence type 398 are potential virulence factors by impacting on the host innate immune response. Frontiers in Microbiology.

[ref-28] Patterson NJ, Werling D (2013). To con protection: TIR-domain containing proteins (Tcp) and innate immune evasion. Veterinary Immunology and Immunopathology.

[ref-29] Radhakrishnan GK, Yu Q, Harms JS, Splitter GA (2009). Brucella TIR domain-containing protein mimics properties of the toll-like receptor adaptor protein TIRAP. Journal of Biological Chemistry.

[ref-30] Rana RR, Simpson P, Zhang M, Jennions M, Ukegbu C, Spear AM, Alguel Y, Matthews SJ, Atkins HS, Byrne B (2011). Yersinia pestis TIR-domain protein forms dimers that interact with the human adaptor protein MyD88. Microbial Pathogenesis.

[ref-31] Rana RR, Zhang M, Spear AM, Atkins HS, Byrne B (2013). Bacterial TIR-containing proteins and host innate immune system evasion. Medical Microbiology and Immunology.

[ref-32] Reder A, Michalik S, Gerth U (2018). How to assess protein stability: half-life determination of a regulatory protein in Bacillus subtilis. Methods in Molecular Biology.

[ref-33] Roy A, Kucukural A, Zhang Y (2010). I-TASSER: a unified platform for automated protein structure and function prediction. Nature Protocols.

[ref-34] Salcedo SP, Marchesini MI, Degos C, Terwagne M, VonBargen K, Lepidi H, Herrmann CK, SantosLacerda TL, Imbert PR, Pierre P, Alexopoulou L, Letesson JJ, Comerci DJ, Gorvel JP (2013). BtpB, a novel Brucella TIR-containing effector protein with immune modulatory functions. Frontiers in Cellular and Infection Microbiology.

[ref-35] Sippl MJ (1993). Recognition of errors in three-dimensional structures of proteins. Proteins.

[ref-36] Sliwoski G, Kothiwale S, Meiler J, Lowe EW (2014). Computational methods in drug discovery. Pharmacological Reviews.

[ref-37] Ve T, Gay NJ, Mansell A, Kobe B, Kellie S (2012). Adaptors in toll-like receptor signaling and their potential as therapeutic targets. Current Drug Targets.

[ref-38] Ve T, Williams SJ, Kobe B (2015). Structure and function of Toll/interleukin-1 receptor/resistance protein (TIR) domains. Apoptosis.

[ref-39] Waterhouse A, Bertoni M, Bienert S, Studer G, Tauriello G, Gumienny R, Heer FT, deBeer TAP, Rempfer C, Bordoli L, Lepore R, Schwede T (2018). SWISS-MODEL: homology modelling of protein structures and complexes. Nucleic Acids Research.

[ref-40] Waterhouse AM, Procter JB, Martin DM, Clamp M, Barton GJ (2009). Jalview Version 2–a multiple sequence alignment editor and analysis workbench. Bioinformatics.

[ref-41] Wiederstein M, Sippl MJ (2007). ProSA-web: interactive web service for the recognition of errors in three-dimensional structures of proteins. Nucleic Acids Research.

[ref-42] Xiong D, Song L, Geng S, Jiao Y, Zhou X, Song H, Kang X, Zhou Y, Xu X, Sun J, Pan Z, Jiao X (2019). Salmonella coiled-coil- and TIR-containing TcpS evades the innate immune system and subdues inflammation. Cell Reports.

[ref-43] Yadav M, Zhang J, Fischer H, Huang W, Lutay N, Cirl C, Lum J, Miethke T, Svanborg C (2010). Inhibition of TIR domain signaling by TcpC: MyD88-dependent and independent effects on Escherichia coli virulence. PLOS Pathogens.

[ref-44] Yang J, Zhang Y (2015). I-TASSER server: new development for protein structure and function predictions. Nucleic Acids Research.

